# VACTERL association complicated with multiple airway abnormalities: A case report

**DOI:** 10.1097/MD.0000000000017413

**Published:** 2019-10-18

**Authors:** Lin Yang, Shu Li, Lin Zhong, Li Qiu, Liang Xie, Lina Chen

**Affiliations:** a Department of Pediatrics, West China Second University Hospital, Sichuan University,; b Key Laboratory of Obstetric and Gynecologic and Pediatric Diseases and Birth Defects of Ministry Education, West China Second University Hospital, Sichuan University,; c Lab of Vascular Remodeling and Developmental Defects, West China Second University Hospital, Sichuan University, Chengdu, Sichuan, Peoples’ Republic of China.

**Keywords:** airway malacia, bridging bronchus, complete tracheal rings, VACTERL association

## Abstract

**Introduction:**

VACTERL association is an acronym that includes vertebral anomalies (V), anal atresia (A), cardiac defects (C), tracheoesophageal fistula (TEF) or esophageal atresia (EA), renal anomalies (R), and limb defects (L). Airway anomalies have rarely been reported with VACTERL association.

**Patient concerns:**

A 10-month-old boy who had been diagnosed with anal atresia and received surgical corrections soon after birth consulted our institution by complaining repeated cough and fever.

**Diagnosis:**

Diagnosis of VACTERL association was finally made. Bronchoscopy and chest CT with computed tomography angiography confirmed multiple airway abnormalities including bridging bronchus, airway malacia, and complete tracheal rings.

**Interventions:**

Supplemental oxygen was provided and antibiotics was initiated.

**Outcomes:**

The patient resolved gradually and was discharged 10 days later. The follow-up showed the patient has remained well just with mild psychomotor retardation.

**Conclusion:**

Multiple airway anomalies may be seen in VACTERL association. It is worthwhile to make special note for evaluating the tracheobronchial pulmonary system by chest CT and bronchoscopy, especially patients presenting with breathing anomalies.

## 1 Introduction

VACTERL association is a condition with multisystem congenital malformations: vertebral anomalies(V), anal atresia (A), cardiac malformation (C), tracheoesophageal fistula (TEF) with or without esophageal atresia (EA), renal dysplasia(R) and limb abnormalities (L).^[1,2]^ Less frequent defects seen with VACTERL association are prenatal and postnatal growth deficiency, laryngeal stenosis, ear anomaly, large fontanels, lower limb defects, rib anomalies, external genital defects, single umbilical artery (SUA), and tethered cord.^[3,4]^ Studies have estimated the frequency of VACTERL association to be between less than 1/10,000 to 1/40,000 infants (approximately <1–9/100,000 infants).^[1]^ Prior studies have estimated that 90% of the patients diagnosed with VACTERL association had three or fewer phenotypes (referred to as VACTERL-like association) and only <1% of patients had all 6 anomalies.^[2,5]^

To our knowledge, airway anomalies have rarely been reported with VACTERL association. We present a patient with VACTERL association found to have multiple airway anomalies—bridging bronchus (BB), airway malacia and complete tracheal rings.

## 2 Case presentation

The patient is a 10-month-old boy, born to Tibetan nonconsanguineous parents. He was admitted to our hospital with cough lasting for 1 week, repeated fever for 2 days. His mother was 28 years old and had an uneventful pregnancy without any risk, such as gestational diabetes, drug intake. No previous pregnancies with congenital malformations were recorded and family history was also unremarkable. He was diagnosed as anal atresia and received surgical corrections of colostomy and posterior sagittal anoplasty for congenital anal atresia soon after birth. Physical examination revealed failure to thrive, right accessory ear, an imperforate anus with a perineal fistula and moist rales in both lungs.

**Figure 1:**
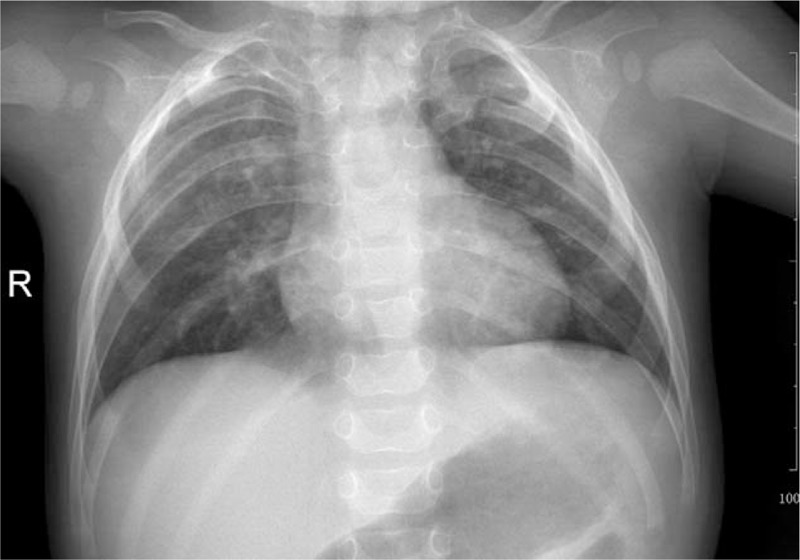
Anterioposterior chest radiograph of the 10-month-old boy with the VACTERL association. The radiograph showed patchy shadows, thoracic vertebral anomalies and rib anomalies (only 10 sets of ribs were seen. The posterior part of the upper ribs was with an irregular shape and the right 2nd, 3rd ribs and the left 2nd rib were short. The upper middle part of the thoracic vertebra was not shown clearly).

**Figure 2:**
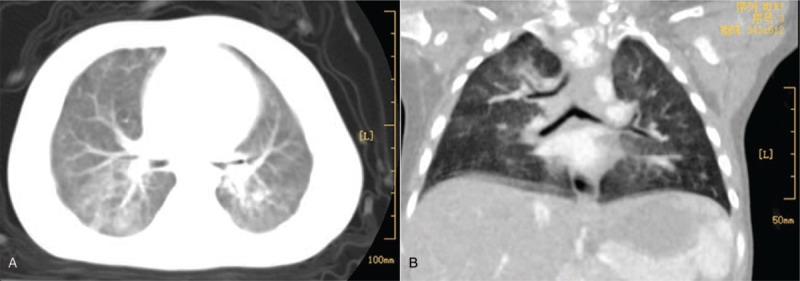
Chest CT with computed tomography angiography of the 10-month-old boy with the VACTERL association. The CT (A) and computed tomography angiography (B) indicated bridging bronchus and tracheal stenosis in the left main bronchus.

**Figure 3:**
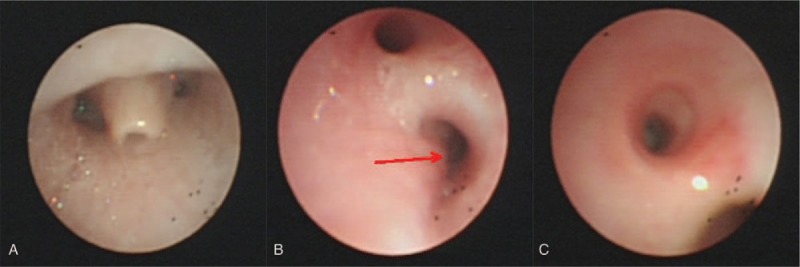
Bronchoscopy of the 10-month-old boy with the VACTERL association. The bronchoscopy showed laryngomalacia (A), bronchomalacia (B) and tracheal stenosis (congenital complete tracheal rings) of the left main bronchus (C).

Complete blood count, coagulation profile, biochemical tests, sputum cultures, and blood cultures were normal. A chest radiograph showed patchy shadows in both lungs, rib anomalies and thoracic vertebral anomalies (Fig. 1). Echocardiography found patent ductus arteriosus (PDA) and a 6-mm atrial septal defect (ASD). As the patient had imperforated anus, rib anomalies, thoracic vertebral anomalies and cardiovascular anomalies, the VACTERL association was diagnosed. Chest CT with computed tomography angiography indicated BB and tracheal stenosis in the left main bronchus. (Fig. 2). Bronchoscopy showed BB, laryngomalacia, bronchomalacia and congenital tracheal stenosis (complete tracheal ring) of the left main bronchus (Fig. 3). Supplemental oxygen was provided and antibiotics was initiated. The patient's respiratory symptoms resolved gradually and was discharged 10 days later. The patient was followed up for 2 year. As the time of writing, the patient has remained well just with mild psychomotor retardation and no longer-term sequelae and recurrent pneumonia. The routine follow-up examinations among blood and urine testing, chest and spinal X-rays, echocardiography and renal function are normal.

Informed written consent was obtained from the patient's parents for publication of this case report and accompanying images.

## 3 Discussion

VACTERL association is a rare and complex condition with highly heterogeneous etiology and manifestations.^[2,6]^ Approximately 90% of VACTERL association cases occur sporadically,^[7]^ with an empiric recurrence risk of 1% or less.^[4]^ Most doctors require at least 3 component features for diagnosis, without clinical or laboratory-based evidence of the overlapping conditions, while others emphasize the presence of certain component features, especially TEF or anorectal malformation.^[1]^ No definite etiology and pathogenesis has been proven, but a defect in mesodermal differentiation due to a variety of causes(genetic, environmental and multi-factors are implicated), in early first trimester (between 6th and 10th weeks of gestation), has been suggested.^[7,8]^

A few airway anomalies have been reported with VACTERL association including tracheal bronchus, ectopic apical segment of right upper lobe bronchus arising from the proximal right main bronchus or from the esophagus, BB, tracheal stenosis, horseshoe lung, and pulmonary agenesis.^[9–11]^ Our patient presented with multiple airway anomalies including BB, airway malacia, and congenital tracheal stenosis (CTS) which had never been reported before. These anomalies are commonly associated with recurrent pneumonia and stridor. Airway malacia is a clinically troublesome condition. The softening of the major airway can lead to symptoms ranging from the minor (harsh barking cough, recurrent chest infections) to severe respiratory difficulties including prolonged ventilator support.^[12]^ BB is an extremely rare congenital anomaly.^[13]^ Gonzalez-Crussi F and colleagues first described BB as a right lower lobe bronchus (often with a middle lobe bronchus) arising from the left main bronchus and bridging the lower mediastinum.^[14]^ CTS is characterized by complete tracheal rings and varies in length, location and severity^[15]^ and is associated with high mortality rates as high as 50%.^[16]^ The formation of complete or near-complete tracheal rings arises from disproportionate growth of the cartilage. CTS can cause varying degrees of respiratory distress, wheezing, coughing, stridor, apnea, cyanosis, and life-threatening airway obstruction. Persistent atelectasis and recurrent pneumonia can also be seen.^[13,15]^

Since the bronchopulmonary foregut malformation (BPFM) gives rise to TEF and EA in VACTERL association, other tracheobronchial pulmonary anomalies may exist.^[2,10]^ It seems that the presence of airway anomalies is underestimated in patients with VACTERL association. This highlights the need to completely evaluate the tracheobronchial pulmonary system in individuals with VACTERL association.^[9]^ Bronchoscopy allows direct visualization of airway anomalies. With respect to some airway abnormalities, such as tracheomalacia and cartilage rings, bronchoscopy persists as the “gold” standard method of diagnosis. It could also provide detailed information about the length, location and severity of tracheobronchial stenosis. When there is clinical suspicion, bronchoscopy is indicated regardless of negative radiologic and clinical findings.^[17]^ Chest CT with computed tomography angiography should been arranged to affirm existence of abnormal vessels and the severity of other lower airway anomalies that could not be found in the bronchoscopy. Surgeons planning to treat such airway anomalies should be aware of the high frequency of abnormal vessels in these cases.^[18]^

Patients and families with features of VACTERL association are told very little about long-term prognoses and outcomes,^[19]^ so the management of patients is complex. According to each component feature of VACTERL association, treatment involves surgical correction of the congenital abnormality and the long-term management.^[1,19]^ In patients with BB, CTS, and airway malacia, no specific therapy is indicated if the patient is asymptomatic. Recurrent pneumonia and localized bronchiectasis are managed with antibiotics, postural drainage and chest physiotherapy. Surgeries are depending on the specific type and clinic symptoms of airway anomalies. Close follow-up and periodic bronchoscopy should be initiated to screen these young patients if they present with persistent respiratory symptoms.^[20]^ Some late-diagnosed malformations resulted in medically significant issues later in life, such as debilitating back pain related to vertebral anomalies, or unilateral renal agenesis with a dysplastic remaining kidney or the presence of a cardiac malformation necessitating careful follow-up of renal or cardiac function.^[1]^ Patients considering VACTERL association undergo testing and/or examination for the presence of each of the core component features, with the following initial testing at a minimum: a thorough history and physical examination by a clinician familiar with the condition, X-rays of the entire spine with consideration of spinal MRI and/or ultrasound, echocardiogram, and renal ultrasound with blood and urine testing for renal function.^[19]^ If available, a pediatrician should follow the infant every 3 to 6 months to monitor signs of impairment and disability, oversee rehabilitation and prescribe adaptive equipment.^[21]^ Besides, clinicians should maintain a low threshold for investigation and further management. The presence of a SUA and certain clues, such as polyhydramnios, lack of a gastric bubble due to TEF, a dilated colon due to imperforated anus on prenatal ultrasound should alert clinicians to the potential existence of tracheal problems.^[1,22]^ It is helpful for appropriate postnatal management.

Because the bifurcation of the tracheal primordium and the subsequent initiation of lung development occur within the period encompassed by the VACTERL sequence, some authors refer pulmonary/tracheal agenesis or tracheal stenosis may be one of the components of the VACTERL association.^[5,23]^ Whether airway anomalies are additional non-VACTERL-type defects or not is still being debated. It is worthwhile to make special note for evaluating the tracheobronchial pulmonary system, especially patients presenting with breathing anomalies. Chest CT and bronchoscopy are indicated in this special group of patients.^[12]^

## Acknowledgments

The authors are grateful to the staff of Department of Pediatrics, West China Second University Hospital of Sichuan University for their work in supporting treatment of the child in this case.

## Author contributions

**Investigation:** Lin Yang, Shu Li.

**Methodology:** Liang Xie.

**Resources:** Lin Yang, Lin Zhong, Li Qiu.

**Writing – original draft:** Lin Yang, Lina Chen.

**Writing – review & editing:** Lin Yang, Shu Li.
